# Regulatory T cells in peripheral tissue tolerance and diseases

**DOI:** 10.3389/fimmu.2023.1154575

**Published:** 2023-05-01

**Authors:** Nardos Cheru, David A. Hafler, Tomokazu S. Sumida

**Affiliations:** ^1^ Department of Immunobiology, Yale School of Medicine, New Haven, CT, United States; ^2^ Department of Neurology, Yale School of Medicine, New Haven, CT, United States; ^3^ Broad Institute of Massachusetts Institute of Technology (MIT) and Harvard, Cambridge, MA, United States

**Keywords:** Regulatory T cells (Tregs), tissue resident, immune tolerance, autoimmune disease, immune interaction

## Abstract

Maintenance of peripheral tolerance by CD4^+^Foxp3^+^ regulatory T cells (Tregs) is essential for regulating autoreactive T cells. The loss of function of Foxp3 leads to autoimmune disease in both animals and humans. An example is the rare, X-linked recessive disorder known as IPEX (Immune Dysregulation, Polyendocrinopathy, Enteropathy X-linked) syndrome. In more common human autoimmune diseases, defects in Treg function are accompanied with aberrant effector cytokines such as IFNγ. It has recently become appreciated that Tregs plays an important role in not only maintaining immune homeostasis but also in establishing the tissue microenvironment and homeostasis of non-lymphoid tissues. Tissue resident Tregs show profiles that are unique to their local environments which are composed of both immune and non-immune cells. Core tissue-residence gene signatures are shared across different tissue Tregs and are crucial to homeostatic regulation and maintaining the tissue Treg pool in a steady state. Through interaction with immunocytes and non-immunocytes, tissue Tregs exert a suppressive function *via* conventional ways involving contact dependent and independent processes. In addition, tissue resident Tregs communicate with other tissue resident cells which allows Tregs to adopt to their local microenvironment. These bidirectional interactions are dependent on the specific tissue environment. Here, we summarize the recent advancements of tissue Treg studies in both human and mice, and discuss the molecular mechanisms that maintain tissue homeostasis and prevent pathogenesis.

## Introduction

Peripheral immune tolerance is crucial to preventing the development of autoimmunity and restraining excessive effector responses in order to protect tissues from immunopathology. One of the critical components of exerting peripheral tolerance is a subset of CD4^+^Foxp3^+^ T cells known as regulatory T cells (Tregs). These cells express the transcription factor Foxp3 which is the master regulator of Tregs and is required for establishing Treg linage and function. Recent studies have described tissue-specific adaptations of a diverse population of immune cells including Tregs. These studies have shown that non-lymphoid tissue Tregs are distinct from their lymphoid tissue (LT) counterparts. For example, non-lymphoid Tregs within tissues such as adipose tissue, colon, skin and lung have different phenotypes from lymphoid Tegs and exert unique functions based on tissue requirements. Moreover, these non-lymphoid Tregs acquire activated/effector phenotypes, tissue homing receptors, and immunosuppressive functionality. Accumulating evidence of non-lymphoid Tregs taken from multiple tissue sites has demonstrated that these tissue-resident Tregs, or tissue Tregs, have shared and/or unique gene expression signatures and that the functional heterogeneity of these Tregs is dependent on their resident tissue environment. The origin of non-lymphoid Tregs, and the differentiation trajectory of Tregs from lymphoid to non-lymphoid tissue, has been studied for several tissue types, and overlapping molecular mechanisms of Treg biology have been identified in mice. Due to the complexity and heterogeneity of tissue Tregs, an in-depth and systematic examination is required to comprehensively understand Treg adaptation to each tissue environment.

The core biology that regulates tissue Treg integrity and homeostasis, and the associated theories, have been based on murine work, and due to the limited accessibility of human tissue Tregs, it has been challenging to translate murine findings to human tissue Treg biology. Complicating matters, studies have shown that there are fundamental discrepancies between human and mice Treg biology. Recent advancements of single-cell transcriptomic and epigenomic technologies have led to novel findings and an expansion of our understanding of tissue Tregs in both human and mice. Here, we review the current understanding of functional and phenotypical heterogeneity of Tregs in the multiple tissue types and discuss the known molecular mechanisms by which Tregs adopt to a specific tissue. We also highlight the differences between tissue Tregs, and note the knowledge gaps between human and mice tissue Treg biology. Although there are other CD4^+^ T cells subsets with regulatory functions, the focus of this review is the biology of Foxp3^+^ Tregs.

## Thymic and peripheral Tregs

The development of Tregs occurs in both the thymus and periphery. Thymus-derived Tregs (tTregs) are mainly mediated by the specificity of T cell antigen receptors (TCRs) to self-antigens. In contrast, peripherally-derived Tregs (pTregs) are differentiated from naïve CD4^+^ T cells into Tregs by acquiring Foxp3 expression in the periphery following recognition of their cognate antigen which is regarded as being non-self (1, 2). To distinguish between tTregs and pTregs, the transcription factor Helios and the membrane protein neuropilin-1 (Nrp1) have been proposed as useful biomarkers, although these are less than optimal in the human setting (3–5). Thus, it is challenging to distinguish between pTregs and tTregs that have been isolated from peripheral tissues.

Given that tTregs are thought to express self-reactive TCRs but not non-self-reactive TCRs, one way to distinguish tTregs from pTregs might be to characterize the TCR repertoire. However, due to the limited availability of such low frequency Tregs, especially in non-lymphoid tissues, it is challenging to determine the antigen specificity of Tregs. Moreover, this hypothesis has been challenged by the observation that tTregs in the colon express TCRs that are reactive to microbiota (6). This could be explained by the cross reactivity of tTreg TCRs to microbial antigens. Nevertheless, it is worth noting that the current dogma asserts tTreg preferentially recognize self-antigens and non-self/foreign antigen specific Tregs are predominantly pTregs (7, 8). Given that it is not always clear to distinguish tTregs and pTregs especially in human, additional studies are needed to clarify the molecular mechanisms that could be used to distinguish pTregs and tTregs during their developmental trajectories.

## Tregs in gut

The frequency of Tregs increases from the duodenum to the jejunum and from the ileum to the colon in mice (9). Interestingly, the frequency of Tregs in different segments of the gut differ between human and mice. In contrast to mice, the Treg frequency in the gut of adult humans increases from the jejunum to the ileum of the colon (10). Of note, this difference was not observed in the human infant gut; Treg frequency in each part of the gut was equally elevated 4 – 6 times higher compared to the frequency of gut Tregs from adult humans (10). These studies highlight the spatio-temporal dynamics of establishing and maintaining Treg pool in the gut (11). Colonic Tregs reside in the lamina propria (LP) under homeostatic conditions, and they preferentially locate in organized lymphoid clusters (12, 13). When mice that have been reconstituted with CD4^+^ T cells specific to ovalbumin (OVA) are challenged with OVA-producing *E. coli*, OVA-specific CD4^+^ T conventional cells (Tconv cells) and Tregs are predominantly observed in lymphoid follicles that contain dendritic cells (DCs) in the lamina propria (14). Notably in patients with inflammatory bowel disease (IBD) and infectious colitis, the number of gut Tregs observed in an inflamed colon are increased and accumulate in lymphoid follicles. The accumulation of Tregs in the lamina propria seems to be a general feature of colon inflammation. These gut-resident Tregs maintain their suppressive function *in vitro* as compared to those isolated from the lamina propria of healthy controls (15–18). The *in vivo* function of Tregs in IBD is likely to be impaired due to the inflammatory microenvironment of inflamed tissue, and the mediated suppression *via* the desensitization of Tconv to Tregs is noteworthy (19, 20). The complex, local microenvironment and cellular interactions contribute to defining Treg functionality in the gut.

### Gut Treg heterogeneity

Tregs in the gut are heterogeneous and several subsets of Tregs have been identified in the intestine and colon. This heterogeneity stems from two major factors: the origin of Tregs (thymic-derived Treg (tTreg) vs. peripherally-induced Tregs (pTregs)), and the expression of phenotypic markers of Tregs. Under homeostatic conditions, the majority of Tregs in the gut are pTregs. The immunoregulatory roles of tTregs and pTregs tend to overlap; however, pTregs play a unique role in the mucosal tissues. Josefowicz et al. generated transgenic mice that were deficient in induced Tregs (iTreg) by deletion of the conserved noncoding sequence-1 (CNS-1) of the Foxp3 gene and demonstrated that these mice exhibit spontaneous Th2 type inflammation in mucosal sites, indicating the importance of pTregs in maintaining mucosal immune homeostasis and highlighting the non-overlapping role of pTregs from tTregs in non-lymphoid tissue (21).

There are at least three major subpopulations of gut Tregs that are differentiated in mice, namely, Gata3^+^ Tregs (~30%), RORγt^+^ Tregs (~40%) and Gata3^-^RORγt^-^ Tregs (~20%) (22, 23). Intestinal Gata3^+^ Tregs have been shown to express high levels of ST2 that is encoded by *IL1RL1.* ST2 is also known as IL-33R and therefore responds to IL-33 which increases expression under inflammatory conditions such as IBD (24). ST2^+^Gata3^+^ Tregs are activated by IL-33 to produce IL-10 and TGF-β (25). This is supported by the fact that Treg-specific Gata3 conditional knockout (cKO) mice develop a spontaneous systemic inflammatory disorder (26) and Gata3 cKO Tregs are defective in their suppressive function and fail to prevent T cell mediated colitis (27). Although Gata3 plays a crucial role in exerting tissue Treg responses during tissue inflammation, Treg-specific Gata3 cKO mice do not exhibit inflammation at steady state, indicating that Gata3 is not required to sustain Treg homeostasis and function at steady state (27).

Sefik et al. and Ohnmacht et al. found that colon Foxp3^+^ Tregs express unexpectedly high levels of RORγt as compared to the other tissue resident Tregs (22, 28). Microbiota-derived signals in antigen presenting cells (APCs) are necessary to induce the differentiation of RORγt^+^ Tregs in the intestine. These RORγt^+^ Tregs constitute ~40-60% of intestine Tregs and predominantly exhibit a Helios^-^ pTreg phenotype. Treg-specific RORγt cKO mice showed a reduced frequency of colonic Tregs, specifically of Helios^–^ pTregs, which in turn resulted in an increased frequency of Helios^+^Gata3^+^ colonic Tregs. Of note, this proportional skewing was not evident in splenic Tregs. There was one phenotypic discrepancy noted between these two studies. Sefik et al. demonstrated that Treg-specific RORγt deletion leads to increased production of both IL-17 and IFNγ but not Th2 cytokines by Tconv cells at steady state. Moreover, trinitrobenzenesulfonic acid (TNBS)–induced colitis in a Th1 and Th17 cytokine-dependent model was significantly exaggerated (11). In contrast, Ohnmacht et al. observed Th2-skewed T cell responses under steady state conditions, which was further enhanced in oxazolone-induced colitis in a Th2 cytokine–driven model. This Th2 skewing phenotype was also suggested by its resistance against helminth infection. In addition, significant Th17 or Th1 cell responses were not evident in a dextran sulfate sodium-induced colitis model (a Th1 and Th17 cytokine-dependent model), which is contradictory to the observation by Sefik et al. Follow-up studies supported the Th17 skewing phenotype in Treg-specific RORγt cKO mice as shown by Sefik et al. using an adoptive T cell transfer colitis model (29). Given that Ohnmacht et al. used Foxp3-GFP-Cre transgenic (Tg) mice from the Jackson laboratory and Sefik et al. and Bhaumik et al. used Foxp3-IRES-YFP-Cre mice from the Rudensky lab, the differential deletion of the Cre line may have caused the phenotypic discrepancy in these studies. Bhaumik et al. further demonstrated that the loss of suppressive function and susceptivity to autoimmune colitis by Treg-specific deletion of RORγt was recovered by an additional Tbet deletion in Tregs, indicating that RORγt-mediated repression of T-bet is critical to the regulation of the suppressive function of colonic Tregs during inflammation (29) (**Table 1**). Taken together, these studies provided important insights into the regulation of the diverse, Treg subpopulations in colon tissue that control intestine inflammation. Their dysfunction could contribute to the development of human IBD.

**Table 1:** The role of core tissue Treg regulatory factors *in vivo* in mice.

	Spontaneous colitis	Steady state Teff phenotype	Steady state Treg phenotype	Treg suppressive function	Foxp3 expression	Treg frequency/number	
Treg GATA3 cKO (26)	Systemic inflammation	Up; IFNγ, IL-4, IL-17	Up: IL-17, IL-21, RORγt^+^ Tregs No change: IFNγ, Tbet	Impaired both *in vivo* and *in vitro*	Impaired	No change in RORγt^+^ Treg Total Treg number was decreased in Sp/LN	(26)Foxp3-GFP-Cre (FGC) Tg mice (Jackson laboratory)
Treg GATA3 cKO (27, 30, 31)	No inflammatory phenotype at young age (developed intestinal pathology and dermatitis after 6 mo. old (31). This was not replicated in (30))	Up: IL-4, ILl-5, IL-13 No change: IL-17 Down: IFNγ in LI-LPL (31)	Up: IL-17 No change: IFNγ, IL-4, IL-5, IL-13 in LI-LPL (31)	Intact *in vitro*	Not affected	No changes in total Treg frequency and numbers in Sp (30) 5-6months old: Total Treg number was increased in LN/mLN but not in Sp (31)	(30, 31)Foxp3-IRES-YFP-Cre mice (Rudensky lab) (27)Foxp3-IRES-Cre mice (Sakaguchi lab)
Treg GATA3/Tbet DKO (30)	Systemic inflammation	Th17 skewed phenotype (adoptive transfer model)	Up : IL-17 No change: IL-4, CD44^+^ activated Treg was decreased in DKO	Impaired *in vivo* (shown by adaptive transfer)	Impaired	Total Treg frequency was down in Sp/mLN RORγt^+^ Tregs were increased in Sp/mLN	Foxp3-IRES-YFP-Cre mice (Rudensky lab)
Treg IRF4 cKO (32)	Systemic inflammation	Th2 up, TNF down	Up: TNF Down: ICOS, MAF, CCR8, *Il10, Il1rl1, Fgl2*, *Gzmb* No change: IFNγ, IL-4, IL-17 CTLA-4, IL2RA, ENTPD1	Intact *in vitro*	Not affected	No change: Total Treg frequency in Sp, Increased total Treg number and frequency in LN	Foxp3-IRES-YFP-Cre mice (Rudensky lab) Increased serum IgG1 and IgE concentration, germinal center formation, and plasma cell tissue infiltration
Treg STAT3 cKO (33)	^+^ (limited to the intestinal mucosa but not systemic)	Th17 (IL-17 and IL-22 were up), not Th1, Th2	Up; *Ctla4, Nt5e, Entpd1*, *Tgfb1* *Down; Il10, Ebi3, Gzmb*, and *Prf1*	Intact *in vitro*, Selectively impaired to suppress Th17 *in vivo*	Not affected	Increased total Treg number and frequency in Sp and mLN, but decreased in draining LN of gastrointestinal tract	Foxp3-IRES-YFP-Cre mice (Rudensky lab)
Treg cMAF cKO (34, 35)	^+^ (with mild signs of immune cell infiltration and tissue destruction)	Th17 skewing	Up: IL-17A and IFNγ after PMA/Iono stimulation. Ki67^+^ Down; *Il10, Ccr2, Ccr5, Areg, Ccr6, Rorc, Il23r, Bcl6, Cxcr5*	Intact *in vitro*, iTreg induction is impaired.	Not affected	No change in frequency of total Tregs and eTreg in Sp/LN/PP/intestine, Significant loss of RORγt^+^Treg in Sp/mLN/PP/siLP/coLP Loss of TFR	Foxp3-IRES-YFP-Cre mice (Rudensky lab)
Treg Blimp1 cKO (36)	Mild colonic inflammation	Up: IL-10, CD44^+^ activated Tconv No change: IL-17	Up: Ki67^+^ activated Tregs, IL-17, No change: IFNγ, IL-4 Down: IL-10	Intact in both *in vitro* and *in vivo* (37, 38)	Not affected	Increased total Treg number and frequency in Li-LP, SP, MLN, No changes in thymus	IL-17 producing RORγt^+^Blimp1^-^ Treg lose suppressive function *in vivo* (transfer model)
Treg RORγt cKO (28)	–	No clear description H. polygyrus infection and oxazolone colitis -> Th2 skewing DSS -> no exaggeration of Th17/Th1 mediated inflammation	Increased frequency of GATA3^+^ Treg	Intact *in vitro*	Not affected		Foxp3GFPCre Tg mice (The Jackson Laboratory)
Treg RORγt cKO (22) (29)	–	Up: IL-17A and IFN-γ after PMA/Iono stimulation.	Increased frequency of GATA3^+^ Treg		Not affected	Decreased Colonic Treg frequency	Foxp3-IRES-YFP-Cre mice (Rudensky lab)

+ means that spontaneous colitis was observed; - means that spontaneous colitis was not observed.

There is emerging evidence that indicates a crucial role of RORγt^+^ Tregs and RORγt^+^ immune cells in the regulation of gut inflammation and tolerance by maintaining the intestinal microbiota balance. However, the detailed molecular mechanisms by which tolerogenic RORγt^+^ Tregs are developed and maintained in the gut remain elusive. Recent studies have uncovered the key pathway governing immune tolerance against microbiota through induction of RORγt^+^ Treg. Furthermore, gut cells that express both MHC-class II molecules and RORγt are thought to be critical to the induction of RORγt^+^ Treg-mediated immune tolerance in gut (39, 40). The MHC-II^+^RORγt^+^ immune cells in the mesenchymal lymph node (mLN) were comprehensively studied using scRNA-seq, and MHC-II expressing lymphoid tissue inducer-like group 3 innate lymphoid cells (ILC3s) were identified as being sufficient to induce RORγt^+^ Tregs in the gut (41–43). These studies shed light on the novel antigen-presenting cells that promote the generation of RORγt-expressing pTregs in the murine gut. Of note, DCs in human fetal gut-draining lymph nodes (44) show a similar gene expression signature as “Thetis” cell subsets (45). Thetis cells are MHC-II^+^ RORγt^+^ APCs that were newly discovered by Akagbosu et al. This discovery indicates a possible overlap between human and mice, at least with some subsets of APCs in gut. Further study is needed to determine if the same cellular interactions are conserved in humans.

### IL-10 is required to maintain colon Tregs

Cytokine IL-10 secreted by intestine Tregs serves a key role by preventing the progression of colonic inflammation, as mice deficient in IL-10 or the IL-10 receptor β-chain (IL-10Rβ) develop spontaneous inflammation of the large intestine. Interestingly, Murai et al. showed that co-transfer of Tregs isolated from *Il10* KO mice can control colitis, indicating Treg derived IL-10 is dispensable for preventing T cell mediated autoimmune colitis. Rather, it was shown that IL-10 secreted by host non-T cells is responsible for preventing colitis (46). Lamina propria macrophage derived IL-10 is required to maintain the Foxp3 expression and suppressive function of Tregs during intestinal inflammation. Notably, in *Il10rb* KO mice, Tregs have normal development and suppressive function; however, *Il10rb* KO Tregs that are transferred into *Rag1* KO mice along with effector T cells cannot prevent colitis. In contrast, another study demonstrated that while *Foxp3^Cre^Il10ra^fl/fl^* mice appeared healthy for up to 8-10 weeks of age, these mice developed clinical symptoms of immune mediated colitis later in life (47). STAT3 phosphorylation on Tregs from *Foxp3^Cre^Il10ra^fl/fl^* mice were impaired compared to control Tregs and IL-6Ra cKO Tregs, indicating that IL-10 signaling on Tregs is important to activate STAT3 under homeostatic conditions. However, the loss of Foxp3 expression on *Il10ra* cKO Tregs was not observed upon co-transfer in the T effector colitis model. Thus, Chaudhry et al. reported that the impaired suppressive function of Tregs without IL-10 signaling is due to the loss of STAT3 activation and not due to the loss of Foxp3 or Treg quantity (33). This discrepancy can potentially be explained by deletion of different IL-10 receptor subtypes (IL-10Ra is specific for IL-10, but IL-10Rb is shared among IL-10, IL-22, and type III IFN).

### Gut Treg repertoire

Tregs under physiological conditions exert TCR-specific suppression of inflammation mediated by other T cell subsets (1, 48). The importance of TCR diversity in Tregs comes from a study showing that Tregs isolated from mice with a limited repertoire are not able to suppress the development of spontaneous, microbiome-dependent, Th17-type intestinal inflammation (49). The colonic Treg population is strongly shaped by the local antigenic milieu and the microenvironment greatly influences Treg cell function and specificity (50–53). Kuczma et al. developed a new approach to examine epitopes of commensal bacteria that are recognized by Treg TCRs and identified *Akkermansia muciniphila* as a microbe that can facilitate Treg induction and expansion of preexisting microbe-specific Tregs (54).

**Figure 1:**
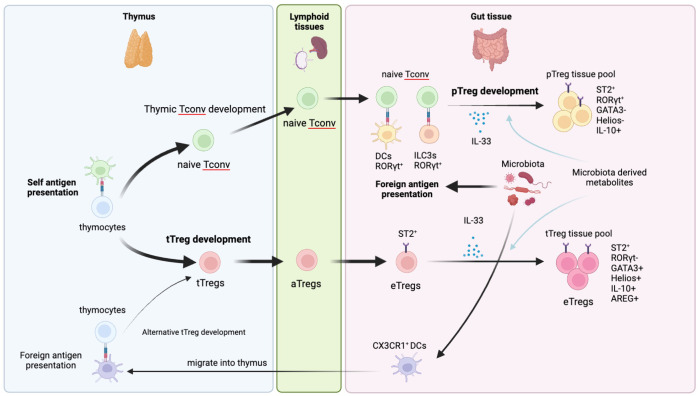
Development of gut tTregs and pTregs. Gut Tregs are composed of tTreg and pTreg pools. tTregs are generally developed in the thymus and preferentially recognize self-antigen. After leaving the thymus, tTregs are activated in secondary lymphoid organs and further differentiate into eTregs in the gut tissue by receiving IL-33 and environmental cues such as microbiota and metabolites. Gut tTregs preferentially express Helios, GATA3 but not RORγt. Recently, an alternative tTreg developmental path has been reported. CX3CR1^+^DCs in the intestine can migrate into thy thymus and present antigens of commensal microbiota to thymocytes to differentiate them into tTregs. Those tTregs harbor TCRs that can recognize foreign antigen and migrate into the gut to establish peripheral tolerance. pTregs are developed from naïve CD4^+^ Tconv stimulated by foreign antigens presented by RORγt^+^DCs and/or RORγt^+^ILC3s in the gut draining lymph nodes and further activated in the gut tissue to acquire suppressive effector Treg features. pTregs preferentially express RORγt but not GATA3 and Helios.

Some overlap of the TCR repertoire between tTregs and pTregs suggested that the ontogeny of gut Tregs is derived from the thymus (6). A recent study by Zegarra-Ruiz et al. demonstrated that antigens of commensal microbiota are delivered by intestinal CX3CR1^+^ DCs and presented to T cells in the thymus (55). This result potentially supports previous findings indicating the importance of the thymic Treg repertoire to the establishment of a tolerogenic Treg pool and maintenance of tissue homeostasis in the gut (6). Furthermore, the time window for presentation of microbial antigens by CX3CR1^+^ DCs in the thymus is limited in early life, which is also seen for the establishment of skin tolerance by Tregs in fetal skin (56). Thus, a similar mechanism could be occurring in skin-thymic communication. While these results are seemingly contradictory to the findings of previous studies claiming the crucial role of the pTreg TCR repertoire that is established locally in the gut (57), both thymic- and peripheral-derived Treg pathways should be harmonized to establish tissue tolerance by Tregs through its temporally and spatially dynamic process (**Figure 1**). Collectively, the thymic and peripheral pathways, along with local antigen presentation by ILC3s, play critical roles in the establishment and maintenance of gut Treg repertoires that generate tolerance in the gut.

## Tregs in adipose tissue

Adipose tissue-resident Tregs have been implicated in the regulation of the immune and metabolic microenvironments of adipose tissue, and therefore implicated in the pathogenesis of obesity-related metabolic disorders. In murine adipose tissue, Th2 and Tregs are the predominant CD4^+^ T cell subsets that maintain homeostatic adipose tissue, together with resident alternatively-activated macrophages (58) and innate lymphoid 2 cells (ILC2s) (59). This regulatory and Th2 skewed immune cell profile in the visceral adipose tissue (VAT) of lean mice is highly reflective of Treg phenotypes characterized by a Th2-like effector Treg signature with high expression of ST2, Gata3, CCR4, Blimp1, KLRG1, and IL-10 (60). The frequency of Tregs within adipose tissue is influenced by several factors, such as obesity, aging, the types and localization of adipose tissue, mouse strains (61), sex (62), and species. The most extensively studied animal for VAT Tregs is the C57BL/6 mouse. Homeostatic, lean adipose tissue of C57BL/6 mouse is enriched with Tregs that are 20-50% CD4^+^ T cells, which is significantly higher compared to the 5-15% make up in the lymphoid tissues of male B6 mice (63–67). VAT Tregs accumulate over time and peak at 24-30 weeks of age in mice. Treg depletion led to skewed proinflammatory metabolic conditions, such as increased fasting blood glucose levels, higher production of proinflammatory cytokines, and impaired insulin sensitivity (64, 68), suggesting that Tregs in adipose tissue regulate the homeostasis of immune balance and metabolism in visceral adipose tissue.

A recent study surprisingly demonstrated the effect of inducible T cell co-stimulator (ICOS) on tissue Treg regulation (69). ICOS KO mice and loss-of-function (LOF) ICOS mutant mice exhibit significant loss of CD44^+^ Tregs in the spleen, pLN, skin, and lungs compared with WT mice. In contrast, Treg abundance in VAT was significantly increased (69). Consistent with this phenotype, ICOS KO and LOF mutant mice are resistant to high fat diet (HFD)-induced insulin resistance. A boosted recruitment of Tregs in VAT is due to the upregulation of CCR3 in the absence of ICOS/PI3K signaling. Further studies with Treg-specific ICOS KO mice are warranted to understand the Treg intrinsic function of ICOS. Moreover, how the PPARγ-mediated, tissue Treg program is modulated by ICOS signaling among different tissues requires further investigation. Another study highlighted the role of insulin signaling and the downstream HIF-1α-Med23-PPARγ-ST2 axis during VAT Treg development in the context of obesity and aging (65, 70). Li et al. demonstrated in lean mice and by age the trajectory of Tregs in VAT where CD73^hi^ ST2^lo^ Tregs differentiated into fat-resident CD73^lo^ ST2^hi^ Treg counterparts. HFD-induced obesity caused premature skewing toward CD73^lo^ ST2^hi^ Tregs, resulting in a diminished pool of CD73^lo^ ST2^hi^ Tregs and a loss in the total number of Tregs. Appropriate insulin signal dynamics was implicated to be important to facilitating VAT Treg differentiation and adaptation (70). Given that multiple signaling molecules are thought to contribute to the tissue residency of Tregs, these studies highlight the complexity of tissue Treg programs. Further study of these tissue-specific Treg programs may provide clues to the perturbation of Treg compartmentalization in a tissue-specific manner, which may potentially allow the introduction of immune tolerance in specific tissues.

### VAT ST2^+^ Treg

The highest levels of ST2^+^ Tregs in C57BL/6 mice were measured in VAT and skin, and the lowest levels in the spleen (67). Notably, ~30% of blood Tregs expressed ST2, which was approximately equivalent to that expressed in lung Tregs but higher than the expression in colon Tregs. The VAT Tregs showed a higher expression of ST2 in male mice than in females, whereas no sex-specific differences were found in the Tregs from the blood, spleen, lungs, colon, or skin (62). Loss of IL-33 or ST2 leads to the dysregulation of homeostatic immune cell balance, characterized by decreased Treg populations in adipose tissue, and manifested in increased weight gain and insulin resistance (71, 72). Systemic administration of IL-33 in mice expanded the pool of ST2^+^ Treg cells and reversed the obesity-induced diminished Treg pool in adipose tissue, resulting in amelioration of hyperinsulinemia and insulin resistance (73). Although the IL-33/ST2 signaling axis was required to promote type 2 cytokine production by adipose tissue Tregs, surprisingly, ST2 expression on Tregs was dispensable for Treg accumulation and maintenance in tissues at steady state (74). ST2^+^ Tregs are developed from precursors found in the spleen and LNs, and a stepwise acquisition of a tissue Treg phenotype is dependent on BATF (23, 75, 76). PPARγ is required to skew tissue Tregs towards a Th2-predominant state and prevent aberrant non-Th2 inflammation (77). IRF4 is also critical to drive the PPARγ-ST2 axis that is essential to facilitate the VAT Treg program and maintain their tissue residency (66, 72). Together, BATF, IRF4, and PPARγ play concurrent roles to promote the VAT Treg program and allow maintenance of VAT Tregs *via* IL-33-ST2 signaling.

### Sex differences in fat Tregs

In terms of resident immune cell populations, it is known that much larger proportions and numbers of Tregs reside in the VAT of male mice as compared to that in age-matched female mice (78). Notably, this sex difference is unique to Tregs, as there were no significant differences between any other major adaptive and innate immune cell populations, including ILC2s (62, 79). Vasanthakumar et al. found that sex hormones shape the sex differences of VAT Tregs and identified Blimp1 as a key regulator of sex-biased enrichment of Tregs in male VAT (58). The observed differences in VAT Tregs was further evaluated in an obese mice by Ishikawa et al. Surprisingly, obesity decreased the population of VAT Tregs in male mice but increased the population in female mice (77). This obesity-induced VAT Treg expansion in females is mediated by estrogen and possibly through CCR6 and CXCR3 but not through ST2. Given the obvious sex bias in obesity and obesity-associated diseases (37), the sex difference in VAT Treg maintenance is of interest, but unfortunately, the current evidence is very limited, especially in humans.

### Obesity and fat Tregs

The VAT in lean mice is enriched with anti-inflammatory immune cells, such as anti-inflammatory macrophages, ILC2s, and Tregs, that keep adipose inflammation in check and thereby maintain tissue homeostasis. Obesity in mice and humans disrupt this homeostatic immune cell balance and cause insulin resistance and adipose tissue inflammation. VAT Tregs, which are highly represented among resident immune cells in lean male, peaking at ~80% of CD4^+^ T cells at 20-30 weeks of age, are diminished by long term, HFD-induced obesity (64, 80). These changes appear to occur *via* reduced local differentiation that is mainly facilitated by PPARγ rather than by impaired homing. Treg-specific depletion of PPARγ can induce VAT-specific loss of Tregs, and administration of a PPARγ agonist can augment VAT Tregs to improve metabolic indices (66, 81). Interestingly, obesity-induced disposition of immune cells in VAT can be imprinted and not easy to reverse, which potentially explains the phenomena that regaining weight further exacerbates the impairment in glucose homeostasis observed with obesity (82). The decreased frequency of VAT Tregs by HFD-induced obesity remains decreased following weight loss and weight cycling (the repeated process of gaining and losing weight) (83). VAT Tregs from obese mice are not only reduced in numbers but also reduced in expression of ST2. Alterations in other IL-33 responsive cell types, such as innate lymphoid 2 cells and mast cells, failed to recover with weight loss and were exacerbated by weight cycling. The intrinsic and extrinsic mechanisms of imprinting the impaired Treg fitness in obese VAD warrants further research.

### Aging and fat Tregs

The accumulation of Tregs in adipose tissue as a function of age was observed in lean C57BL/6 mice, but not in obese mice (65, 81, 84). Age-associated insulin resistance (IR) and obesity-associated IR are two physiologically unique forms of IR that are driven by distinct mechanisms. Bapat et al. found that mice deficient in fat Tregs are protected against age-associated IR yet remain susceptible to obesity-associated IR and metabolic disease. By contrast, selective depletion of fat Tregs *via* anti-ST2 antibody treatment increased adipose tissue insulin sensitivity. Interestingly, this age associated enrichment of VAT Tregs is not coordinated with ILC2s which has been shown to support IL-33 mediated Treg expansion in VAT (85). A recent study found numerical and functional loss of ILC2s in aged adipose tissue and increased expression of IL-33 in aged VAT tissue (86), indicating that homeostatic interaction between ILC2s and Tregs is disrupted by aging. Currently, the mechanistic insights for age related expansion of tissue Tregs are poorly understood.

### Adipose Treg repertoire

VAT Tregs have a highly restricted distribution of TCR sequences and exhibit distinguishable TCR repertoires from that of their counterparts in the spleen and lymph nodes (64). Furthermore, in Vα2-Vβ4 VAT-Treg TCR transgenic mice, the frequency and total number of Tregs are specifically elevated in VAT, but not in the spleen (87). Moreover, VAT Tregs depend on the recognition of antigen(s) presented by MHCII on APCs for their retention and accumulation in VAT (88, 89). However, the specific antigen(s) recognized by VAT Tregs remains unknown. The discovery of VAT Tregs as key players in the maintenance of tissue homeostasis opened the door for a new paradigm where Tregs control the tissue integrity and keep inflammation in check, thereby preventing aberrant inflammation-mediated metabolic disorders. A better understanding of the ontogeny and TCR repertoire of VAT Tregs, and elucidating the complex interplay of VAT Tregs with stromal cells in different contexts, is required to fully understand the Treg program in VAT. Additionally, exploring human VAT Treg biology is critical for translational Treg research and for reducing the knowledge gap created by mouse-dominated studies.

## Tregs in skin tissue

The skin is a barrier that protect organisms from external physical injury and pathogen invasion. The roles of Tregs at barrier surfaces such as intestine, lung, and skin are critical to maintaining tissue homeostasis and restoring normal function after insult. The Tregs at these barrier surfaces vary in their features. For example, a notable difference between intestinal- and skin-resident CD4^+^ Tregs is the ratio of pTregs and tTregs. Further, a signature feature of gut Tregs is that a majority are RORγt^+^ Helios^-^ peripheral Tregs that have been induced by commensal microbes. By contrast, RORγt^+^ Tregs are a minor subpopulation (~5%) of skin Tregs in lean mice (90). Skin Tregs preferentially express high levels of GATA3 (50-60% of skin Tregs) which skews the skin Treg transcriptomic signature toward Th2 as compared with Tregs in LN or in other tissues such as lung and colon (23, 67, 91). DNA methylome and chromatin accessibility analysis of Tregs from multiple mouse tissues identified epigenetic differences between VAT and skin Tregs, and a Th2 skewed transcriptional signature of VAT- and skin-Tregs. This obvious Th2 bias was not observed in human VAT- and skin-Tregs (92, 93). Rather, BATF seems to be a common regulator of the tissue Treg programs in both human and mice (38). In homeostatic skin Tregs, less than 10% of Tregs express Nrp1 (94), which is supported by a lower expression of both NRP1 and IKZF2 in skin Tregs and colon Tregs as compared to LN and spleen Tregs in mice (23). This shows that Foxp3^+^ Tregs in homeostatic skin could primarily be peripheral Tregs. In contrast, human skin Tregs express higher levels of both *NRP1* and *IKZF2* as compared to blood Tregs. Further studies are needed to better understand how homeostatic skin Treg pools are maintained in both human and mice.

The establishment of homeostatic skin tolerance relies on Treg migration from the thymus. This is supported by evidence of an abrupt accumulation of activated Tregs that have migrated from the thymus during the early neonatal stage in mice (56). This large Treg influx is unique to skin tissue, and the influx is not observed in skin-draining lymph nodes or in the intestinal lamina propria (where the Tregs consists of only 10-15% of CD4^+^ T cells (95)). Treg pool expansion in the skin in early life was significantly interfered by sphingosine-1-phospate receptor antagonist FTY720 which blocked the egress of lymphocytes from the thymus and LNs, and resulted in a failure to establish tolerance to cutaneous microbes. After the acute influx of Tregs into neonatal skin, Treg frequency gradually decreases with age, from a peak of ~80% in neonates to a low of ~50% in adult mice (56). Given that spleen residing Nfil3^+^Klrg1^+^ tissue-precursor Tregs can also migrate into the skin in adult mice (75), the skin Treg pool in later murine life could be supplied from splenic Treg precursors, which potentially explains the lower frequency of Nrp1^+^ Tregs in adult skin. These murine phenomenon was also observed in human fetal skin (95, 96).

### Hair follicle stem cells and Tregs

**Figure 2:**
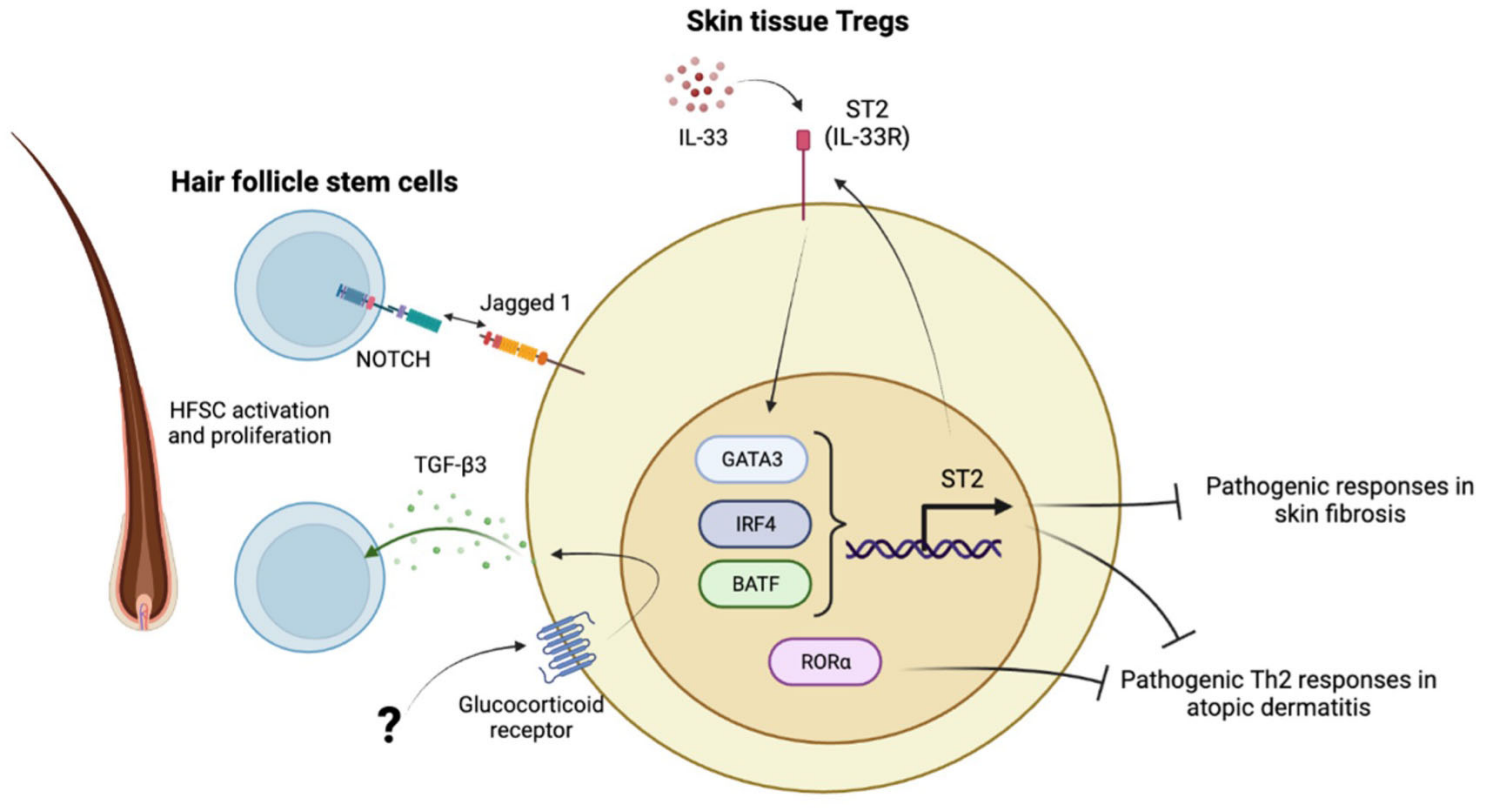
Characteristics of skin tissue Tregs. IL-33-ST2 axis maintains skin Treg signature with high GATA3, IRF4, BATF expression. RORα is highly expressed in skin Tregs. Those transcription factors are critical to provide skin immune tolerance and prevent development of skin fibrosis and atopic dermatitis. The other aspect of skin Tregs is to maintain hair follicle homeostasis through interaction with hair follicle stem cells (HFSCs). Skin Tregs activate and facilitate HFSCs proliferation through Jagged 1-Notch axis and glucocorticoid receptor mediated TGF-β3 secretion. The upstream mechanisms how GR is activated in the hair follicle is still not known.

Recent studies demonstrated a non-canonical function of Tregs that is independent of immune tolerance involving maintenance of the hair follicle stem cell (HFSC) niche (97, 98). Ali et al. first described the unique role of skin resident Tregs during HFSCs response to hair removal by bolstering HFSC activation to facilitate hair regeneration (97). Tregs in skin tissue predominantly reside proximal to the hair follicles and promote HFSC proliferation and differentiation. Mechanistically, the authors found Jagged 1 [encoded by *Jag1* and known to act as Notch ligand (99)] is highly expressed in skin Tregs compared to Tregs in skin draining LNs and is responsible for HFSC activation potentially through Jag1-Notch pathway activation (97). In another study by Zheng et al. the authors identified that Tregs in hair follicle express higher glucocorticoid receptor (GR) compared to splenic Tregs and demonstrated that GR expression on Tregs is necessary for hair removal induced HFSC proliferation and hair regrowth (93). GR signaling induced TGFβ3 expression, but not TGFβ1 nor TGFβ2, in Tregs and this GR-TGFβ3 axis in Tregs is responsible for HFSC activation, thus providing another pathway that plays a critical role in Treg-HFSC crosstalk to maintain hair follicle response in skin (**Figure 2**). Given that immune cells, including Tregs, are able to communicate with tissue stem cells to facilitate tissue repair and maintain tissue homeostasis (100, 101), further studies focusing on Treg – stem cell crosstalk in tissue will unveil non-canonical Treg function.

### Skin fibrosis and Tregs

Acute Treg depletion induces fibroblast activation and leads to fibrotic changes in the skin. When Tregs were chronically depleted, the skin tissue developed fibrosis and bleomycin-induced skin fibrosis was exaggerated (91). Mice lacking ST2 specifically on regulatory T cells showed significantly worse skin fibrosis, increased Th2 to Treg ratio, and increased IL-13 expression in the skin compared to wild-type mice (102), indicating that Tregs play a critical role in preventing an excessive Th2 response in the skin and the development of skin fibrosis. In systemic sclerosis (SSc) patients, while the proportion of Foxp3^+^ skin Tregs was not changed between SSc vs. healthy individuals, the Th2 cytokine production in skin SSc Tregs was significantly increased as compared to skin Tregs isolated from healthy individuals (103). MacDonald et al. further demonstrated that ST2 expression in skin Tregs from SSc patients was also increased and possibly enhanced Th2 cytokine production in SSc Tregs through IL-33. Notably, neither a Th1- or Th17-associated surface phenotype or cytokine production was altered in SSc skin Tregs. This human study supports the murine observation that the ST2-Th2 axis serves as a key pathway in skin Treg promoting skin fibrosis, whereas there are some contradictory results showing enriched Treg infiltration in the skin from early SSc patients (104). This contradictory result may reflect different sample collection times during progression of fibrosis and disease.

In addition to GATA3 (105) and IRF4 (32), a recent study identified a Treg intrinsic factor that regulates aberrant Th2 skewing in mice. Malhotra et al. found that transcription factor RORα is highly expressed in skin Tregs unlike in Tregs originating from the blood or LNs in both human and mice (106). This was confirmed by another study using scRNA-seq (23). Targeted deletion of RORα in mouse Tregs did not affect Treg frequency but increased IL-10 production in skin Tregs at steady state. In a MC903-induced atopic skin model, Treg-specific RORα cKO mice showed exaggerated atopic histological changes and eosinophilia infiltration of the skin. An increase in CCR3 and Th2 cytokine production and a decrease in expression of *CCR6, TNFRSF25* (DR3), and *CD73* were observed, indicating that RORα in skin Tregs suppresses IL-4 expression and enhances expression of DR3 which is the receptor for the tumor necrosis factor (TNF)-like cytokine 1A (TL1A) that promotes Treg function (106). Given that increased RORα expression in tissue Tregs is a shared feature of skin and colon Tregs (23) and that TNF-associated receptors and pathways are enriched in tissue Tregs (23), the TNFRSF-RORα and TNFRSF-NFκB axes could serve as core pathways for tissue Treg maintenance. Taken together, skin Tregs constitute a large portion of resident T cells and govern skin homeostasis through complex interactions with stromal cells and cutaneous bacteria on the skin. A comprehensive approach to dissect these interactions should provide a better understanding of the Treg-centric, tolerogenic program in the skin. Notably, the ontogeny of skin Tregs is relatively unexplored as compared to that of colon Tregs. Given the multiple commonalities between skin and colon as barriers against microorganisms, further studies of skin Treg ontogeny should be informative.

## Tregs in lung tissue

Tregs play a dual role of promoting or inhibiting pulmonary inflammation, allergies, and fibrosis. This duality stems from the complex steps of developing chronic pulmonary diseases; Tregs act differently in different phases of pulmonary inflammation and different Tregs subtypes have different roles in the progression of pulmonary pathology (107). In patients with idiopathic pulmonary fibrosis (IPF), no difference was found in the frequency of total CD4^+^CD25^+^Foxp3^+^ Tregs in circulation among patients with IPF, primary Sjögren’s syndrome-related interstitial pneumonia (pSS-IP), or healthy controls (HCs). However, Treg subtypes were significantly skewed towards activated Tregs (aTregs) from resting Tregs (rTregs) in IPF patients compared with pSS-IP or HCs. Importantly, in the bronchial alveolar fluid (BALF) from patients with IPF, increased proportions of aTregs were reported (108). In contrast, Tregs in both BALF and peripheral blood were reduced compared with those of healthy volunteers and patients without IPF (109).

Anti-CD25 antibody-mediated Treg depletion has been studied in multiple, fibrotic lung inflammation models in order to determine the contribution of Tregs to lung inflammation. The amelioration of disease by Treg depletion was observed in bleomycin-induced pulmonary fibrosis (by inducing Th17 and other CD4^+^T cell subset responses in the lungs) (110), in irradiation-induced pulmonary fibrosis (by inhibiting the epithelial-to-mesenchymal transition in lung epithelial cells) (111), and in silica-induced lung fibrosis (112). In contrast, Treg depletion exaggerated 1,3-β-glucan-induced lung inflammation (113) and beryllium oxide-exposed HLA-DP2 Tg mice showed exacerbated lung inflammation and enhanced granuloma formation (114).

In patients with asthma, the Th17/Treg balance was skewed toward Th17 (115) and Treg frequency was decreased in BALF of asthmatic children compared with that of control subjects. Moreover, inhaled corticosteroid treatment was associated with increased percentages of Tregs in both peripheral blood and BALF (116). Overall, the accumulating evidence indicates that Tregs play critical roles in the suppression of allergic inflammatory responses and airway hyperreactivity, mostly *via* the IL-10 dependent pathway (117–119).

### Tissue resident lung ST2^+^Treg

Tissue Tregs are required to prevent acute lung injury induced by intratracheal (i.t.) bleomycin injection. Local delivery of IL-33 at the time of acute lung injury is protective but requires the presence of Tregs that secrete IL-13 in response to IL-33. Lung Tregs express high levels of ST2 and upon IL-33 stimulation both mouse and human Tregs secrete IL-13. This induction of Treg IL-13 is required to prevent the accumulation of proinflammatory monocytes at injured tissue sites, and to protect mice from fatal lung injury (120).

ST2 expression on lung Tregs is regulated by the balance of Bcl6 and Blimp1 (121). Blimp1 is required to induce ST2 expression in lung Tregs in a cell-intrinsic fashion, which is counter regulated by Bcl6. Of note, Bcl6-deficient ST2^+^ Tregs promote airway inflammation in the context of house dust mite-induced allergic airway inflammation, indicating that Bcl6 is necessary for the suppressive function of ST2^+^ Tregs. Kanno et al. focused on the effect of fatty acid metabolism on lung Tregs and found that the administration of IL-33 enhanced the acquisition of fatty acids from the environment in lung Tregs, especially in ST2^+^ lung Tregs. Through transcriptomic and proteomic analysis of Tregs, they found that acyl-coA synthetase bubblegum family member 1 (ACSBG1) was highly enriched in ST2^+^ lung Tregs and was a metabolic regulator of effector Tregs. ACSBG1-dependent mitochondrial fitness in Tregs is critical for tissue ST2^+^ Treg function and the resolution of lung inflammation (122).

### IL-4R signal and lung Tregs

**Figure 3:**
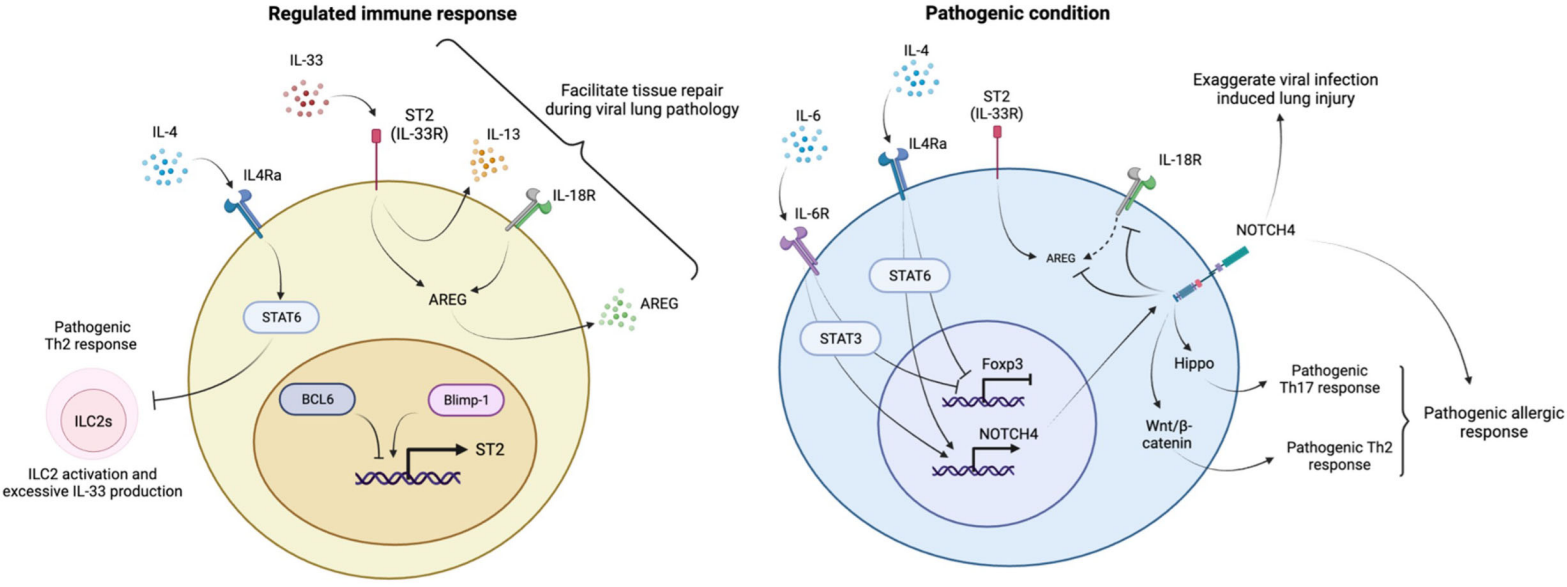
Regulation of lung homeostasis by lung Tregs. The IL-33-ST2 axis induces IL-13 and AREG to prevent inflammatory responses and facilitate tissue repair during respiratory viral infection. ST2 expression in lung Tregs is controlled under the balance between Blimp1 and BCL6. The IL4R-STAT6 pathway is necessary to keep ILC2s in check to prevent aberrant activation and excessive production of IL-33. Under inflammatory conditions, IL-4 and IL-6 suppress Foxp3 expression and induce NOTCH4 expression in lung Tregs. NOTCH4 enhances Th17 and Th2 pathogenic responses during allergic inflammation through activation of Hippo and Wnt/β-catenin signaling respectively. NOTCH4 also suppresses IL-18R/AREG axis and reduce AREG production, leading to progressive viral infection mediated lung pathology.

An enhanced IL4Rα-STAT6 pathway in CD4^+^ T cells impairs iTreg differentiation and promotes conversion of naïve CD4^+^ T cells toward a Th2 or Th17 cell fate (123, 124). IL-4 mediated impairment of Treg induction is also known to be STAT6 dependent (78), and HDAC9 is involved in chromatin modifications by regulating histone acetylation at the Foxp3 locus (125, 126). To study the role of IL-4 signaling on Tregs in the context of allergic inflammation, Khumalo et al. generated Treg specific IL-4Rα cKO mice and interrogated lung Treg function in the HDM-driven airway inflammation model (127). Genetic deletion of IL4Rα in Tregs resulted in impaired expansion and functionality in the local lung tissue during airway inflammation. Khumalo et al. further identified that type 2 cytokines and IL-33 production by ILC2s were exacerbated in the lung tissue of mice with targeted deletion of IL-4Rα in Tregs, indicating that IL-4-responsive Tregs regulate IL-33 production and ILC2 activation in the lung. Consistent with the outcomes of this study, Noval Rivas et al. reported that IL-4 production by IL-33-stimulated ILC2s blocks the generation of allergen-specific Tregs and promotes food allergy (128). Therefore, novel strategies to block ILC2 activation or the IL-33/IL-33 receptor pathway can lead to innovative therapies in the treatment of food allergy (128). These studies highlight the close interaction between ILC2s and lung Tregs during type 2 inflammation, and show that the IL-33/ST2 signaling axis is critical to controlling allergic inflammation by lung Tregs (**Figure 3**).

Harb et al. identified Notch4 as being highly inducible in allergen-specific Tregs during allergic inflammation. The induction of Notch4 expression by IL-6, followed by Wnt and Hippo pathway activation in pulmonary-induced Tregs, subverts their stability and functions to promote allergic asthma. Harb et al. further determined the downstream pathways of Notch4 that contribute to the conversion to Th2 or Th17 cytokine–producing ex-Tregs and found that the Hippo pathway controls the Th17 arm and that canonical Wnt (β-catenin) controls the Th2 arm (129). Recently, Benamar et al. found an induction upstream of Notch4 by studying the asthma susceptible IL4R576 allele in humans and showed a positive association with asthma severity and Notch4 expression on T cells, including Tregs. They further demonstrated that IL-4Rα signaling upregulated Notch4 expression in lung Tregs and its downstream mediators, Yap1 and β-catenin, in ovalbumin-driven allergic airway inflammation in IL4Rα^R576^ mice. The upregulation of Notch4 was dependent on growth factor receptor-bound protein 2 (GRB2) and IL-6 receptors on lung Tregs (130). Notch4 expression on lung Tregs is also associated with lung pathology in the context of viral infection, including COVID-19 disease. Notch4 expression on Tregs limits the important role of the IL18Ra/amphiregulin (AREG) axis in tissue repair by Tregs during lung inflammation (131). ST2 expression on Tregs, which marks it as an effector subset of suppressive tissue Tregs, was not linked with Notch4-mediated impairment of the IL18Ra-AREG axis (132), indicating a dispensable role of the IL33-ST2 axis on Notch4^+^Tregs in respiratory viral infection where anti-viral type 1 IFN response is the dominant pathway (**Figure 3**).

### Heterogeneity of lung Tregs

Lung Tregs are increased under various inflammatory conditions and consist of heterogeneous subpopulations. Ichikawa et al. examined the role of lung resident CD4^+^ T cells, including Tregs, in a murine model of chronic lung inflammation induced by repeated exposure to *Aspergillus fumigatus* (*A. fumigatus*) (133). In this model, T cells play a critical role in the development of lung pathology (T cell-deficient Foxn1nu mice develop limited lung inflammation and fibrosis). Blood-circulating CD4^+^ T cells were labelled by intravascular injection of CD4-PE antibodies so that tissue-localized cells and blood-borne cells could be distinguished by flow cytometry. Tissue resident Tregs were marked by Foxp3^+^CD44^hi^CD69^hi^CD4-PE^neg^ and primarily belonged to a CD103^hi^ tissue resident CD4^+^ T cell fraction (a 60-80% CD44^hi^CD69^hi^CD4-PE^neg^CD103^hi^ subset comprised of Foxp3^+^ tissue resident Tregs, along with a 5-10% CD44^hi^CD69^hi^CD4-PE^neg^CD103^lo^ subset). This tissue resident CD103^+^ Treg expansion was also observed in beryllium-induced lung inflammation (114, 134). A majority of those tissue resident Tregs are RORγt^+^ (~50%) or GATA3^+^ (~10%) Tregs, and a limited fraction of Tregs express both RORγt and GATA3, which is consistent with colon Tregs [Helios expression was not assessed in the study (133)]. Surprisingly, Treg specific RORγt deletion resulted in a minor impact on lung inflammation in the *A. fumigatus* infection model, indicating that Foxp3^+^RORγt^+^ Tregs might not contribute to lung pathology driven by repetitive infection of *A. fumigatus* in the lungs. This limited contribution of RORγt^+^ Tregs to lung inflammation contrasts with the requirement of RORγt^+^ Tregs for regulating gut inflammation. To better understand the role of RORγt^+^ Tregs in lung tissue, further investigations of the molecular mechanisms of RORγt^+^ Treg development during lung inflammation is needed, as is the exploration of their topology.

## Tregs in central nervous system

The brain has been historically described to be an immune privileged site inert to foreign grafts; however, unless animals were immunized prior to a graft, there was no immunological response (135). Recent work has since shed light on the interactions of the CNS with the immune system as well as defined brain-resident adaptive immune cells (136, 137). Among the immune cells found within the CNS, brain Tregs have been identified at considerable levels in rodents, with Tregs accounting for 7.5% of brain CD4^+^ T cells in adult mice and Foxp3^+^ cells accounting for 15% of rat cerebral CD4^+^ T cells (138, 139). There is an analogous distribution of CD4^+^ T cells in healthy human brains obtained from temporal lobe surgery and in brain lesions of multiple sclerosis (MS) patients, with Tregs making up 10-30% of CD4^+^ T cells (138, 140). With age, the frequency of Tregs in the meninges and deep cervical lymph nodes increases. This is attributed to the loss of CCR7 on meningeal Tregs that prevents migration and promotes retention. Mice lacking CCR7 showed a similar phenotype to aged mice with increased meningeal Tregs and poor cognitive function. Anti-CD25 treatment of aged mice specifically reduced meningeal Tregs and resulted in improved cognitive performance (141). A vast majority of brain Tregs expressed Helios, suggesting that they were of thymic origin and similar to resident Tregs in VAT and skin (88, 106, 142–144). Of these cells, a large fraction was CD95^hi^CD45RO^hi^ as compared to blood peripheral Tregs, indicative of susceptibility to apoptosis (145). Brain resident Tregs acquire a mature and residency phenotype as compared to peripheral Tregs. For example, 80% of cerebral Tregs express markers of memory and activation as compared to 13% of splenic Tregs (138, 139). Compared to splenic T cells, both conventional T cells and Treg brain resident cells expressed elevated RORγt, and Tregs specifically showed more GATA3 expression during ischemia (142, 143). This is consistent with an activated phenotype as GATA3 is upregulated upon TCR ligation, independent of Th2-like cytokines, and has been implicated as a necessary factor of tissue resident Tregs to maintain their identity and prevent acquisition of an effector phenotype (27). In MS, the percentage of activated peripheral Tregs are elevated whereas the resting Treg fraction dropped in numbers in comparison to healthy donors. Peripheral Tregs from patients with Alzheimer’s disease (AD) have been shown to be either comparable in frequency to those of healthy controls or less, depending on which Treg surface markers were used in the study. However, there is consensus that Tregs from AD patients have a larger CD25^dim^ population that is associated with a resting phenotype as the Tregs have reduced suppressive capacity (146, 147). In an examination of HLA-DR as a marker of maturation and activation, MS Tregs, rather than AD Tregs, had a higher expression of HLA-DR, specifically in the secreting and activated subsets (147). These data indicate that CNS resident Tregs are present and, as with other tissue resident cells, express an activated phenotype compared to peripheral Tregs.

Tregs have been found to be in close proximity to microglia throughout different regions of the brain, particularly in the meninges. Compared to circulating and splenic Tregs, brain Tregs have a higher expression of genes for neuropeptide Y (NPY) and serotonin receptor 7 (HTR7). Stimulation with HTR7 specifically promoted brain Treg proliferation and not splenic Treg proliferation. HTR7 deficient Tregs failed to proliferate and resulted in worsened neurological outcomes (142). This may be attributed to HTR7 involvement in the activation and proliferation of CD4^+^ T cells *via* activation of ERK1/2 and NF-κB (148). *In vitro* suppression assays with LPS-stimulated microglia/macrophage cultures found that cerebral Tregs are better than splenic Tregs at suppressing pro-inflammatory cytokine expression. This may be due to greater expression of IL-10 and CTLA-4 amongst CNS Tregs compared to splenic Tregs. However, there was no observable differences between the two Treg compartments in suppression of stimulated conventional T cells (139).

### Neuronal cells - Treg interaction

**Figure 4:**
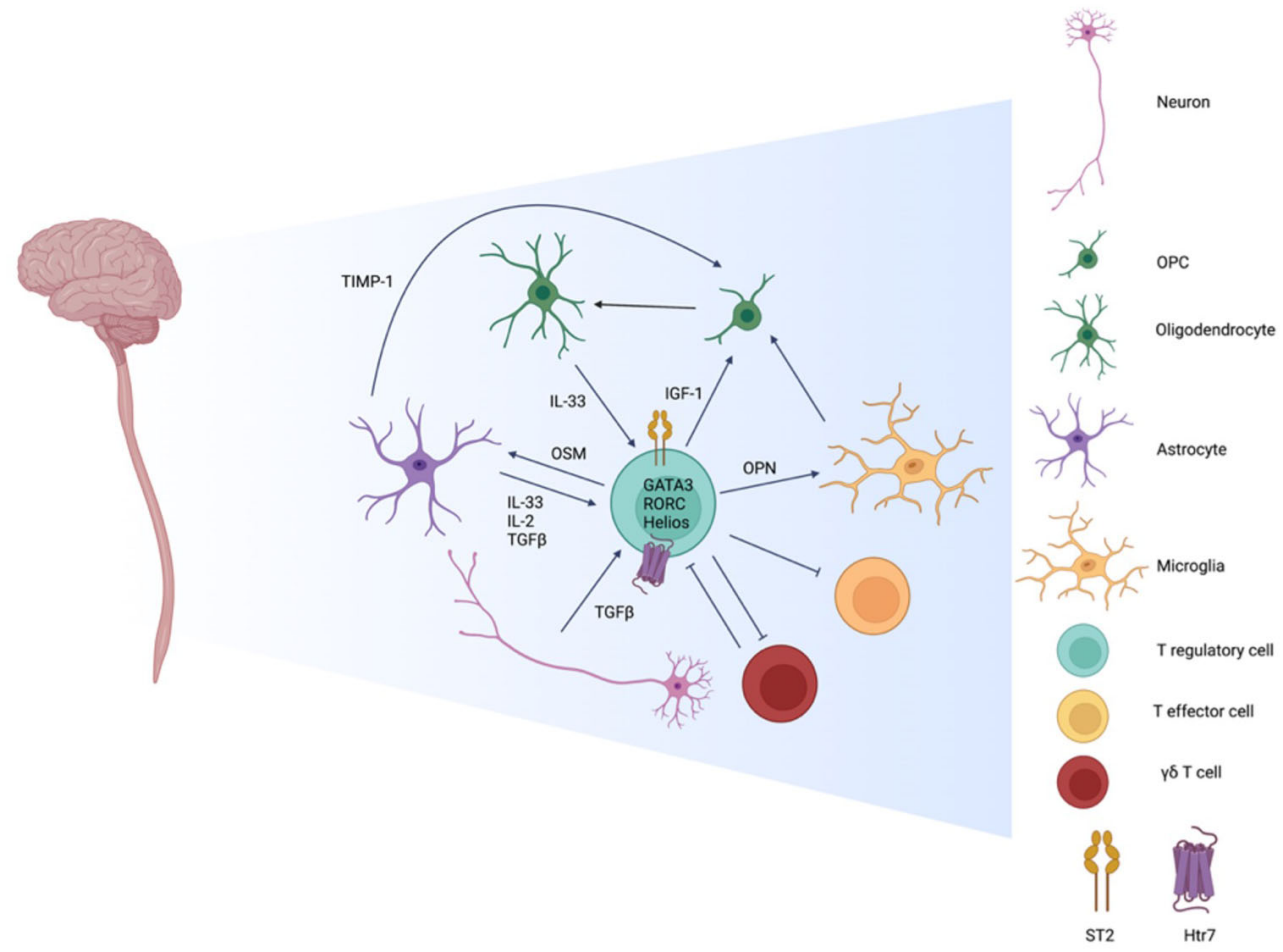
Interactions amongst Tregs and cells residing in the CNS. CNS Resident Tregs are primarily of thymic origin noted by Helios expression and have a tissue specific profile marked by comparatively elevated expression of the IL-33 receptor ST2, the serotonin receptor Htr7, and the transcription factors GATA3 and RORC. While CNS resident Tregs have higher expression of activation markers, in comparison to peripheral Tregs, they are as efficient in suppressing T effector cells. During inflammation and disease, inflammatory γδ T cells are one of the first immune infiltrates and shape the initial phase of insult by suppressing the Treg pool. At the resolution phase, the roles reverse as Treg numbers expand while γδ T cells diminish. CNS resident Tregs adopt a non-immune role of shaping the environment by promoting remyelination in varies capacities. The differentiation of oligodendrocyte precursor cells (OPCs) to mature oligodendrocyte is influenced directly by Treg derived insulin like growth and factor 1 (IGF-1). Indirect means include Treg priming of microglia and astrocytes by osteopontin (OPN) and oncostatin M (OSM), respectively, to promote OPC differentiation *via* tissue inhibitor of metalloproteinases-1 (TIMP-1). Neurons and glial cells also regulate Tregs by secreting IL-33, IL-2, and TGF-β to support local Tregs function and identity.

A dissection of the interactions between glial cells and brain-resident Tregs has revealed that resident Tregs, like oncostatin M (OSM) and insulin like growth factor 1 (IGF-1), are involved in oligodendrocyte precursor cell (OPC) differentiation (143). OSM acts through its receptor expressed on astrocytes to promote expression of tissue inhibitor of metalloproteinases-1 (TIMP1), which then aids in remyelination by promoting OPC differentiation (149). IGF-1 receptor signaling in OPCs and mature oligodendrocytes are necessary for their differentiation and regeneration, respectively (150). In a co-culture system with Tregs, microglia cells upregulate an anti-inflammatory program and genes that promote brain repair such as vascular endothelial growth factor A (VEGFA) and transglutaminase 2 (Tgm2), which has been previously reported to be involved in oligodendrogenesis. Treg-derived osteopontin (OPN) act on microglia cells to enhance secretion of factors involved in OPC differentiation (143). Along with possessing a higher expression of activation markers, brain resident Tregs acquire a unique tissue-specific profile that is associated with their function. Overall, brain resident Tregs have a non-immune role in supporting remyelination by producing factors important for oligodendrocyte differentiation and regeneration *via* indirect and direct modes (**Figure 4**).

Emerging studies have shown that Tregs and neurons can interact in a direct and indirect manner. Indirectly, addition of Treg-conditioned media to co-cultures containing OPCs and dorsal root ganglion neurons resulted in enhanced myelination. This effect is attributed to Tregs promoting OPC differentiation *via* Treg-derived cellular communication network factor 3 (CCN3) (151). CCN3 in pancreatic beta cells has been shown to be under control of Foxo1, a key transcription factor for Treg identity and function (152, 153). While CCN3 is primarily expressed by neurons in the CNS and has been shown to be involved in OPC differentiation, there is debate regarding the requirement of CCN3 for myelination or remyelination (154). A direct relationship between neurons and Tregs has been demonstrated to center around TGF-β that is produced by neurons in the presence of T cells along with the upregulation of co-stimulatory B7.1, B7.2, and ICAM-1 in IFNγ and TNFα dependent manners. While neurons are not classical APCs due to the lack of expression of MHC class II molecules, direct contact between T cells and neurons resulted in augmented T cell proliferation and expression of TGF-β1 and CTLA-4. When co-cultured with neurons, encephalitogenic T cells upregulated Foxp3 and CD25. CD25^+^ TGFβ1^+^CTLA-4^+^ T cells generated from the neuronal co-cultures were able to suppress proliferation of activated T cells. In an adoptive-transfer model of EAE, encephalitogenic T cells were supplemented with Tregs derived from neuron co-cultures and resulted in a mitigated induction of EAE (155). Additionally, astrocytes produced TGFβ and uniquely IL-2. This was illustrated by the maintained frequency of Foxp3^+^ Tregs in astrocyte co-cultures, whereas Foxp3 expression was lost when Tregs were cultured alone. This effect seems to be Treg specific as co-cultures of T conventional cells with astrocytes did not result in a change of Foxp3 expression. However, culturing T conventional cells and Tregs with astrocytes reduced apoptosis. This can be attributed to IL-2 signaling of Tregs as there is enhanced phosphorylation of STAT5 in co-cultured Tregs. As previously discussed, Tregs promote remyelination of neurons by inducing OPC differentiation, whereas neurons, along with astrocytes, serve to promote and sustain Treg identity. The cross-talk between neuronal cells and CNS Tregs act in concert to ameliorate disease and further closes the gap between the central nervous and immune systems (**Figure 4**).

### IL-33/ST-2 axis in CNS Tregs

The IL-33-ST2-AREG axis may also play a pivotal role in the interactions between Tregs and glial cells. Studies in cancer mouse models revealed that IL-33 from the tumor microenvironment initiates ST2 signaling in Tregs, as well as positively regulating ST2 expression, resulting in enhanced suppressive function, greater AREG production, and increased tumor number and size (156, 157). AREG, a key molecule in tissue repair, can also act in a feedforward fashion to further improve Treg function (132, 158). Data from EAE and ischemic brain injury models show a similar axis in which IL-33 expression is enriched in the CNS and upregulated in oligodendrocytes and astrocytes (140, 142). It has also been demonstrated that brain Tregs express ST2 and AREG, in which ~60% of Foxp3^+^ cells express ST2 within MS active lesions (138, 140). In perturbing the axis, Tregs deficient in *Il1rl1* were unable to proliferate or suppress astrogliosis. Similarly, AREG deficient Tregs had a parallel phenotype and was rescued with intraventricular administration of exogenous AREG in a mouse model of ischemic stroke (142). ST2 deficient Tregs at steady state did not have altered tissue accumulation; however, the defect was evident during EAE as there was enhanced infiltration of Tregs to the CNS. Additionally, there is an enlarged IL-17^+^ γδ T cell population suggesting that despite the increase of Tregs, ST2 is required for anti-inflammatory activity and for managing γδ T cells that is concomitant with enhanced disease severity (74). While the CNS-specific, soluble factors that are involved in the cross-talk between Tregs and γδ T cells have yet to be fully elucidated, in the context of allergy ST2^+^ Tregs modulate γδ T cell pool *via* IL-35 secretion in an IL-33 dependent manner (159). Further work has investigated the interaction of γδ T cells and Tregs within the CNS. IL-23 is a key cytokine in brain inflammation that accumulates in the CNS during EAE (160). Interestingly, IL-23 can directly modulate Treg function by suppressing ST2 expression, thereby ablating IL-33 responsiveness and functionality (25). Cytokine IL-23 has been implicated in regulating Tregs indirectly *via* IL-23R^+^ γδ T cells. As the number of IL-23R^+^ γδ T cells in the CNS decreased, there was enhanced infiltration of Tregs and increased suppressive function. Notably, T-cell receptor delta chain (*Tcrd*) deficient mice had increased CD103^+^ Treg infiltrates and reduced EAE severity*. In vitro* assessment using supernatant from IL-23-stimulated γδ T cells revealed that naïve CD4^+^ T cells were less able to be skewed towards Treg lineage when cultured with TGF-β. This was attributed to IL-6 and IL-21 independent suppression of Foxp3 promotion by TGF-β. This supports a model in which IL-23R^+^ γδ T cells destabilizes Foxp3 expression and thereby perturbs Treg function (161). These studies propose an axis in which alarmin IL-33, made by glial cells in response to insult, act on ST2^+^ Tregs to enhance their regulatory function and control the diametric dynamic of γδ T cells and Tregs during disease.

## Treg ontogeny from secondary lymphoid tissues to non-lymphoid tissue

Even in unchallenged homeostatic condition, Tregs in lymphoid tissues such as LNs and spleen do not exclusively exhibit a naïve state. Some Tregs already exhibit an activated and effector phenotype, though at lesser extent than Tregs from non-lymphoid tissue. While the entire mechanism on how homeostatic activation of Tregs are regulated in either an antigen specific or non-specific fashion remains unclear, recent studies suggest that Tregs in both lymphoid tissue and non-lymphoid tissue are important to establish peripheral tolerance. For instance, antigen presentation in secondary lymphoid organs is necessary for homeostatic maintenance of antigen-specific pTregs to constrain antigen-specific immunity (156). Recently, Gu et al. nicely demonstrated that gut Treg induction against *Helicobacter hepaticus (Hh)* in colonic immunity is dependent on secondary lymphoid organs (mesenteric lymph node and caecal patch) but not sufficient to confer effector Treg suppressor function by using lymphotoxin−α deficient mice where secondary lymphoid organs were lacking (157). They also found that the gut tissue is the dominant site for Hh specific Tregs to become fully activated and acquire suppressive effector function. These studies indicate that lymphoid tissue and non-lymphoid tissue play independent roles in induction and maturation of Tregs in the gut.

After the identification of the unique characteristics of VAT Tregs with high expression of PPARγ, the ontogeny of VAT Tregs has been further explored. Based on studies with VAT specific TCR transgenic mice, Li et al. proposed a stepwise Treg differentiation trajectory from lymphoid tissue to VAT Tregs, highlighting a subpopulation of PPARγ^lo^ splenic Tregs as a precursor of VAT Tregs (87). This led to a scRNA-seq study to reconstruct the Treg differentiation trajectory in pseudotime space based on gene expression signatures of Tregs from both lymphoid tissue (LT) and non-lymphoid tissue (NLT) (23, 75, 162). These studies further support a stepwise Treg differentiation theory and indicate that tissue Treg precursors in lymphoid tissue that are marked by PPARγ expression or by the primed tissue Treg transcriptional signatures have the potential to differentiate into not only VAT Tregs but also into multiple tissue Tregs. Parabiosis experiments revealed that there are relatively low exchanges of VAT Tregs with the circulating splenic progenitor Tregs as compared to the exchanges of splenic Tregs (88). This indicates that local expansion, along with supplementation from the circulation, shapes the Treg pool in non-lymphoid tissue such as VAT and gut. Although PPARγ expressing progenitors have been shown to differentiate into multiple tissue Tregs in mice, the Treg intrinsic factors that influence tissue residency are poorly understood.

Recent study by Yang et al. defined three Treg states in secondary lymphoid tissues in mice by focusing on TCF1 and LEF1 expression; quiescent resting Tregs (rTregs: CD62L^+^CD44^neg^TCF1^+^), activated Tregs (aTregs: CD62L^-^CD44^int/hi^TCF1^+^), and effector Tregs (eTregs: CD62L-CD44^hi^TCF1^-^) (163). They proposed a model of peripheral, but not thymic, Treg differentiation, in which TCF1 and LEF1 are necessary for homeostatic maintenance of the aTreg pool that is critical to generate Tfr in order to restrain Tfh and GC B cell response. By contrast, eTregs (TCF1^-^ subset) in secondary lymphoid tissues could be the precursors of tissue eTregs as proposed by a stepwise Treg differentiation (163). Future studies are needed to determine how PPARγ and TCF1/LEF1 expression are linked or independently regulated in secondary lymphoid tissue Tregs.

**Figure 5:**
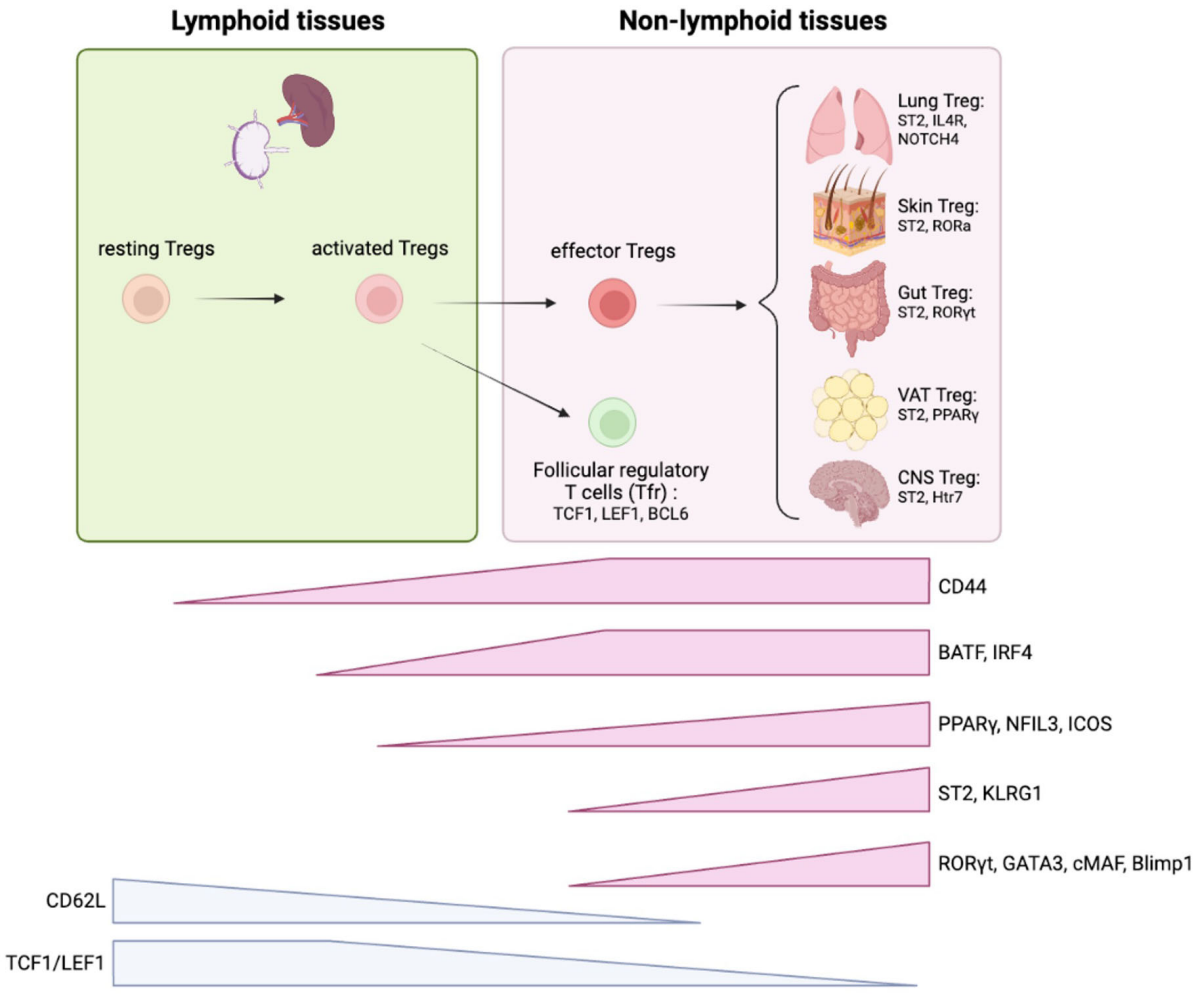
Treg ontogeny and key signature factors of tissue Tregs. Tregs are initially developed in thymus and migrate into secondary lymphoid organs. TCF1/LEF1 are critical to maintain quiescent state of Tregs. Once resting Tregs are activated, the program initiated by BATF/IRF4 facilitates effector Treg differentiation. The stepwise induction of PPARγ and NFIL3 further proceed this program to achieve effector Treg signatures (i.e. ST2, KLRG1, ICOS). Effector Tregs migrate into non-lymphoid tissues and adopt to the microenvironment where they reside. ST2 expression is a general tissue Treg signature that is required for homeostatic maintenance of the tissue Treg pool through the IL-33/ST2 axis. Each tissue Tregs exhibit unique features. Lung Tregs express higher IL4R and Notch4; Skin Treg express higher RORα; Gut Treg express higher RORγt, VAT Treg express higher PPARγ, and CNS Treg express higher Htr7. These features are mostly based on mice studies.

These evidence provide a novel aspect of extrathymic Treg differentiation from lymphoid tissue to non-lymphoid tissue and highlights the importance of studying the trajectory of different Treg pools in lymphoid tissues to better understand tissue Treg ontogeny (**Figure 5**).

## Tissue Tregs in autoimmune diseases

### Tregs in inflammatory bowel disease/Crohn’s disease

Studies in mice and humans have highlighted the critical role of regulatory T cells in immune homeostasis and disease. In Crohn’s disease, there are defects in the number and suppressive function of regulatory T cells, while in inflammatory bowel disease, the distribution of Tregs and their ability to traffic to the GI tract are impaired. Maul et al. found that the Treg pool in the intestinal lamina propria of patients with active IBD was significantly decreased as compared to that of diverticulitis patients (18). However, the abundance of human RORγt^+^ Tregs is comparable between healthy individuals and patients with inflammatory bowel disease (164). Given the immunosuppressive function of Tregs in a model of T cell-mediated colitis in rodents and the potentiation of Tregs to induce or promote transplantation tolerance (165), a clinical trial with a single infusion of freshly isolated peripheral blood-derived Tregs to a patient with active Crohn’s disease (CD) was safely conducted without significant side effects (166, 167). By contrast, a high number of human RORγt^+^ Tregs were found in colitis-associated dysplastic areas than in non-dysplastic areas from individuals with ulcerative colitis (168). Rizzo et al. investigated the role of Tregs in colitis-associated colorectal cancer (CAC) using the azoxymethane and dextran sodium sulfate-induced CAC model. They demonstrated that Treg-specific RORγt cKO mice are resistant to tumor development without affecting the grade of inflammation in the tumor microenvironment, indicating that RORγt^+^ Tregs facilitate CAC development independent from regulating colonic inflammation. RORγt^+^ Tregs are also required to suppress Foxo3 in tumor-infiltrating DCs, leading to the establishment of a IL-6 rich, pro-tumor microenvironment. Interestingly, mice colonized with IBD microbiotas had fewer colonic RORγt^+^ Tregs than mice colonized with healthy donor microbiotas. The mice colonized with IBD microbiotas exhibited more severe disease than those colonized with healthy donor microbiotas in the T cell transfer colitis model (169). Of importance, the IBD microbiota-mediated reduction of tolerogenic RORγt^+^ Tregs is reversed by the transplantation of healthy donor-derived microbiotas (170). These data indicate the dual functions of RORγt^+^ Tregs in the gut, namely, the regulation of IBD-related inflammation through tolerogenic potential, and by contrast, the facilitation of CAC development by RORγt^+^ Tregs.

Given that gut homeostasis is built on the complex interplay between diverse commensal microbiota and immune cells, including Tregs, therapeutic perturbation of the microbiota and immune cells should be well considered and tailored depending on the course of disease and magnitude of inflammation. In addition, the diverse genetic background of humans is another component that cannot be perfectly modelled by murine studies. Genome-wide association studies identified hundreds of susceptible loci for IBD/CD (171, 172); however, our understanding of those genetic risks to gut Treg mediated tolerance is highly limited. Thus, further studies examining human gut Treg biology is required to further develop therapeutic strategies for IBD/CD.

### Tregs in RA synovial tissue

Rheumatoid arthritis (RA) is a chronic autoimmune disease which is characterized by leukocyte infiltration into synovial tissue followed by cartilage and bone damage. Collagen-induced arthritis (CIA) is the most widely used animal model of RA. In mice with CIA, CD25^lo^Foxp3^+^CD4^+^ T cells lose Foxp3 expression and acquire a Th17-like signature. These exFoxp3 Th17 cells accumulate in inflamed joints and contribute to the induction of osteoclasts which leads to osteoclastic pathology (173). A potential limitation of the CIA model is that its pathogenicity relies heavily on autoantibodies against type II collagen as demonstrated by the induction of disease through serum transfer from arthritic mice. Thus, the model does not mimic T cell-mediated autoimmune responses of human RA. By contrast, self-reactive CD4^+^ T cells, but not autoantibodies, cause arthritic inflammation in the joints of SKG mice (174).

The frequency of Tregs in peripheral blood of RA patients is controversial. The majority of reports show that the percentage of circulating Treg is decreased (175–177), while other reports demonstrate elevated (178) or similar (179) levels in RA patients in comparison to healthy donors. A meta-analysis of 31 studies applying a stricter definition of Tregs as CD25^high^/Foxp3^+^ supported the decreased frequency of blood Tregs in patients with RA (180). The degree of Foxp3^+^ Treg inhibitory activity of RA patients during the remission of disease was higher than that of active RA patients (181). Moreover, an inverse correlation between Treg inhibitory activity and disease activity score 28 (DAS28) in patients with moderate and high disease severity was observed. These data indicate that higher Foxp3^+^ Treg inhibitory activity reflects the restoration of peripheral T cell tolerance in patients with active RA, suggesting Treg suppressive function *in vitro* as a prognostic marker of RA disease activity. In contrast, IL-17 producing CD161^+^ Tregs are shown to be increased in RA peripheral blood and their frequency was negatively correlated with disease activity (182).

Both blood and synovial fluid Tregs have been studied in humans. Most reports show that Foxp3^+^ Tregs infiltrate inflamed joints (183, 184). Tregs from synovial fluid express higher levels of CTLA-4, GITR, OX40, and Foxp3 and are less responsive to proliferation under TCR stimulation, indicating a terminally-differentiated phenotype. Of note, Tregs from diseased joints are potently suppressive *in vitro* (185–187), although a subpopulation of Tregs secrete IL-17A (188). In oligoarticular juvenile idiopathic arthritis (oligo JIA), which is the most common form of chronic inflammatory arthritis in children, synovial Tregs exhibit lower Helios and higher CTLA-4 with a Th1 skewed signature (higher CXCR3/CD161/IFNγ/Tbet), but not a Th17 skewed signature (IL-17/STAT3/RORγt/CCR6) (189). Jule et al. further demonstrated that synovial CXCR3^+^ Tregs maintained their suppressive function and lineage-defining methylation patterns, suggesting a comparable Treg identity as blood Tregs. Other Treg subpopulations were identified as HLADR^+^ and CD161^+^ Treg clusters.

In another study by Rossetti et al. (190) they identified activated HLA-DR^+^ Tregs in the blood of juvenile idiopathic arthritis (JIA) patients and found suppressive Tregs that can recirculate between the systemic circulation and the synovial microenvironment. The blood HLA-DR^+^ Tregs showed a suppressive function that was comparable to other blood Tregs and maintained a Treg-specific demethylated region. Importantly, TCR repertoire analysis revealed that blood HLA-DR^+^ Treg clones are more closely related to synovial Tregs than to the other circulating Tregs. A recent study using scRNA-seq with TCR repertoire analysis demonstrated that one of the clonally expanded T cells in synovial fluid is Tregs (191). The clonally expanded Tregs expressed higher *CCR8 and CXCR6* which is consistent with the tumor infiltrating tissue Treg signature (110, 192, 193). Lutter et al. performed scRNA-seq with TCR analysis of synovial fluid Tregs (sf Tregs) from JIA patients and identified four Treg clusters: incoming recently-arrived Tregs that expressed *CCR7, KLF2, TCF7 and LEF1* (37.24% of sf Tregs); effector Tregs with a dominant suppressive profile that expressed *TIGIT, CTLA4, IKZF2, LAYN* (31.22% of sf Tregs*)*; effector Tregs with a cytotoxic profile that expressed *LGALS1, CXCR6, CCR5, TNFRSF8, GZMA* (23.57% of sf Tregs); and GPR56^+^CD161^+^CXCL13^+^Tregs that expressed *CXCL13, GPR56, MYO7A, BHLHE40, PTPN13 and KLRB1* (7.96% of sf Tregs) (194). The GPR56^+^CD161^+^ CXCL13^+^ synovial fluid Tregs were unique with their high CXCL13 expression as compared to peripheral blood Tregs. These Treg subpopulations exhibited relatively lower Helios expression as compared to other GPR56^-^CD161^-^Tregs from either the blood or synovial fluid Tregs, which is consistent with another study of synovial fluid Tregs from two patients with HLA-B27^+^ ankylosing spondylitis and three patients with psoriatic arthritis (195). These Tregs maintained higher Foxp3 expression as compared to blood Tregs, but a relatively lower Foxp3 expression than GPR56-CD161^-^ synovial fluid Tregs. Moreover, results from an *in vitro* suppression assay indicated that GPR56^+^CD161^+^ synovial fluid Tregs are proficient suppressor cells that are as proficient as other synovial fluid Tregs. TCR repertoire analysis demonstrated that the three effector Treg populations share a similar TCR repertoire, but the GPR56^+^CD161^+^CXCL13^+^ synovial fluid Treg cluster represented a relatively unique TCR repertoire compared to the other two effector Treg subsets. In addition, there was a mere overlap between Treg vs. non-Treg clones in synovial fluid CD4^+^ T cells, suggesting minimal *in situ* differentiation of CD4^+^ T cells to Tregs for any of the synovial fluid Treg clusters.

Based on these observations, it is expected that further studies using single-cell level TCR repertoire analyses of synovial fluid and blood Tregs would uncover details of clonal overlap between these Tregs. This could potentially reveal the antigen responsive Treg clones that contribute to shaping the inflammatory tissue microenvironment. Given that TCR repertoires are largely private across individuals, which is consistent with the mechanisms underlying HLA-driven positive selection of newly rearranged TCRs, the identification of arthritis-specific clonotypes of Tregs will require TCR repertoire assessment on an individual basis. As such, the investigation of both blood and synovial Tregs focusing on HLA-DR^+^ Tregs would facilitate the investigation of Treg biology at the level of individual RA patients.

### Tregs in systemic lupus erythematosus

Studies on the role of Tregs in lupus have revealed conflicting results, suggesting both increased and decreased presence of Tregs during disease (196–199). A meta-analysis evaluated 18 published studies of blood Treg frequency, absolute numbers, and suppression capacities in lupus patients (a total of 628 patients and 601 healthy controls). Due to a high degree of heterogeneity, a random effects model was used to assess the mean differences between active SLE and controls. Overall, and although the data was extensively heterogeneous, the percentages of Tregs in active SLE patients were significantly lower than those in controls. By contrast, Guo et al. recently demonstrated that Treg frequency is increased in SLE blood using both scRNA-seq and flow cytometry in a relatively larger cohort (HC = 30, SLE = 44) (200). Two Treg clusters were identified and marked by the expression of CCR7^+^ (the naïve Treg-like cluster) and by CD74^+^ (the effector Treg-like cluster). The frequencies of both Treg clusters were increased in patients with SLE compared to healthy controls. Of note, the effector Treg-like cells in SLE were capable of producing more effector cytokines such as IFNγ, IL-2, and TNFα than the effector Treg-like cells of healthy controls, suggesting that SLE Tregs exhibit an exhaustion-like signature with potentially impaired suppressive function. Hanaoka et al. demonstrated that an increase in circulating SLE Treg frequency is paralleled by an increased Systemic Lupus Erythematosus Disease Activity Index score and downregulation of Foxp3, but with increased expression of CD161/CD49d. Production of IL-17 was upregulated but there was no change in IFNγ production or suppressive capacity in an *in vitro* suppression assay (201). Consistent with these results, recent studies using Helios as an additional functional Treg marker showed that FoxP3^+^Helios^+^ Treg cell numbers are elevated in patients with clinically active SLE (202, 203).

Frantz et al. investigated the number of Tregs in skin specimens obtained from patients with various subtypes of cutaneous lupus erythematosus (CLE) by immunohistochemical analysis. The number of Foxp3^+^ Tregs in CLE was significantly reduced, but there was no correlation between disease subtype and the frequency of Foxp3^+^ Tregs in the skin of patients with CLE. In peripheral blood, no significant differences were observed in the number of Tregs. Recent advancements in scRNA sequencing enabled a study of tissue infiltrating- and resident-Tregs from skin and kidney tissues affected by SLE (204, 205). Dunlap et al. identified Treg clusters in both skin and kidney tissues; however, and unexpectedly, the transcriptional changes between Tregs from cutaneous lupus skin and healthy skin were very moderate. Due to the limited number of cells that were isolated from tissues and the relatively lower frequency of Tregs among the isolated infiltrating immune cells from inflamed tissues, the number of Tregs analyzed by scRNA sequencing was too low to provide detailed cellular characteristics of infiltrating Tregs in SLE patient tissue. Further studies that include a focus on infiltrating CD4^+^ T cells are warranted to decipher the features of tissue Treg in SLE patients.

## Concluding remarks

A critical role of the immune system is to mount immune responses to foreign microbial antigens while suppressing responses to autoantigens. Complex and harmonized interactions between innate and adaptive immunity are necessary to achieve tolerance. Recent advancements of immunobiology research sheds light on the role of the immune system that confers tissue tolerance not only in lymphoid tissues but also in peripheral, non-lymphoid tissues. Tregs have emerged to be crucial to governing peripheral tissue tolerance. Disruption of Treg-mediated tolerance in tissues can result in a wide range of human disorders. While there seems to be core biological programs determining the trajectory of Treg development from the thymus to peripheral tissues, Tregs residing in each tissue exhibit unique signatures that could be acquired through interactions with stromal cells in resident tissues. Conversely, Treg function can be highly affected by their pathogenic microenvironment which could trigger dysfunctional behavior of Tregs that may facilitate immune pathologic responses. Thus, not only is understanding Treg intrinsic biology important but also achieving a comprehensive view of Treg interactions with its partners is required for a complete picture of Treg-mediated peripheral tolerance in homeostasis and disease settings. Moreover, it is challenging to study the functions of tissue resident Tregs in humans. Given the non-negligible differences between mice and human Tregs, further studies exploring the function and biology of human tissue Tregs are key to developing novel therapeutic approaches that manipulate Treg function.

## Author contributions

NC and TS wrote the manuscript with input from all authors. TS and DH conceived the study and were in charge of overall direction and planning. All authors contributed to the article and approved the submitted version.
